# Sequence-assignment validation in protein crystal structure models with *checkMySequence*

**DOI:** 10.1107/S2059798323003765

**Published:** 2023-06-14

**Authors:** Grzegorz Chojnowski

**Affiliations:** a European Molecular Biology Laboratory, Hamburg Unit, Notkestrasse 85, 22607 Hamburg, Germany; MRC Laboratory of Molecular Biology, United Kingdom

**Keywords:** macromolecular crystallography, register shifts, *findMySequence*, model validation, *checkMySequence*, sequence validation

## Abstract

Sequence-register shifts remain one of the most elusive errors in experimental macromolecular models. They may affect model interpretation and propagate to newly built models from older structures. In a recent publication, it was shown that register shifts in cryo-EM models of proteins can be detected using a systematic reassignment of short model fragments to the target sequence. Here, it is shown that the same approach can be used to detect register shifts in crystal structure models using standard, model-bias-corrected electron-density maps (2*mF*_o_ − *DF*_c_). Five register-shift errors in models deposited in the PDB detected using this method are described in detail.

## Abstract 2

It is shown that *checkMySequence*, an automated method for validating sequence assignment in cryo-EM structures of proteins, can be used to validate crystal structure models.

## 1. Introduction

Macromolecular crystallography (MX), nuclear magnetic resonance (NMR) and, more recently, cryogenic electron microscopy (cryo-EM) are the methods of choice for detailed analysis of the structures of proteins and their complexes. Over five decades, the efforts of generations of structural biologists using these methods have resulted in the deposition of over 200 000 macromolecular structures in the Protein Data Bank (PDB; wwPDB Consortium, 2018 ▸), most of which (87%) have been solved by MX. The PDB is an invaluable resource of experimentally determined structures for half of the known protein families (according to InterPro version 92; Paysan-Lafosse *et al.*, 2022 ▸), often in multiple biochemical contexts and conformations, in the apo form and with natural or artificial interaction partners. Recently, it has enabled the training of artificial intelligence (AI) tools that have extrapolated the available experimentally determined structural information to virtually any known protein sequence (Jumper *et al.*, 2021 ▸; Baek *et al.*, 2021 ▸).

Despite the unquestionable value of the accumulated knowledge, the PDB is also known to contain models that are overall or partly incorrect. The issue of error propagation from PDB models to AI-based methods remains an open question (Jones & Thornton, 2022 ▸). Currently, it seems that AI-predicted models are an excellent aid in building and correcting experimental structures (Terwilliger *et al.*, 2023 ▸). However, structures that can be validated experimentally constitute only a tiny fraction of the almost 200 million predicted models that are already available in the AlphaFold2 database (Varadi *et al.*, 2022 ▸). Therefore, the importance of extensive validation of both newly determined experimental models and those already available in the PDB, for which experimental data are available (86% of MX structures), cannot be overemphasized, as they provide the most reliable and detailed source of information on macromolecular structures currently available.

Errors in experimental macromolecular models have become easier to detect over time due to the continuous development of model-validation tools. The cross-validation in macromolecular crystallography (splitting reflections into ‘free’ and ‘work’ sets) introduced in the early 1990s helps to avoid gross errors in models (Brünger, 1992 ▸). Local tracing errors can usually be identified as a poor fit between an atomic model and the corresponding combined electron-density map or prominent difference-density map peaks. Although the maps are calculated using phases derived from a tentative model, which may hinder the detection of errors, the refinement programs account for this using σ_A_ weighting of map coefficients, which reduces model bias (Read, 1986 ▸). The local quality of models is validated using expert systems such as *PROCHECK* (Laskowski *et al.*, 1993 ▸), *WHAT_CHECK* (Hooft *et al.*, 1996 ▸) and *MolProbity* (Prisant *et al.*, 2020 ▸) focused on the stereochemical plausibility of model coordinates. Multiple map-based and geometry-based validation approaches can also be conveniently used, for example, in *Coot* (Casañal *et al.*, 2020 ▸) during the interactive model-building process to identify and correct errors. Finally, detailed validation precedes the deposition of models in the PDB, which is nowadays an indispensable part of the peer-review process in most scientific journals (Gore *et al.*, 2017 ▸).

Indeed, with the availability of a wide range of model-validation techniques, the overall quality of structural models has improved significantly (Brzezinski *et al.*, 2020 ▸). It has been observed, for example, that the ‘clashscore’ from the *MolProbity* suite, a sensitive indirect indicator of tracing and map-fit issues, has been steadily improving over time for PDB depositions (Williams *et al.*, 2018 ▸). At the same time, however, the common usage of Ramachandran plot restraints in model building and refinement often masks model issues, making a lack of unusual torsion angles in PDB depositions a weak indicator of a high model quality (Sobolev *et al.*, 2020 ▸). This may be confusing to structural biologists, especially those new to the field. They frequently struggle to distinguish outliers from errors and to choose the optimal refinement strategy. I have observed that this often results in reducing the final model refinement to the improvement of PDB ‘sliders’, which graphically combine several global model-quality indicators, which may obscure real, local problems.

One of the most elusive errors in macromolecular models are register shifts, where the backbone is traced correctly but the residues are systematically assigned the identity of a residue a few amino acids up or down in sequence (Wlodawer *et al.*, 2018 ▸). This issue can be easily detected in high-resolution structures as it causes significant mismatches between the model and the electron-density map for several neighbouring side chains. Moreover, prominent difference-density peaks indicate missing or excess side-chain atoms in the model. At lower resolutions, however, a deteriorated map–model fit due to incorrectly modelled side chains can easily be mistaken for poorly resolved model fragments. Difference-density peaks are usually weaker and visible only for a few well resolved side chains. The effect on global model–data fit scores (*R*_free_ or the *R*_work_/*R*_free_ gap) can be detectable but is typically small as the number of excess or missing atoms is usually negligible compared with the overall size of the model. Register shifts resulting from a tracing error (a deletion or insertion) can be detected by the presence of backbone geometry outliers. For example, deletions are often compensated with a stretched backbone, which after refinement may result in Ramachandran plot outliers and twisted peptide bonds (clearly marked with a yellow polygon in *Coot*). Wrongly assigned side chains are often in nonrotameric conformations and result in severe steric clashes that cannot be corrected during refinement. Finally, series of side chains without matching density may also locally reduce the map–model correlation coefficient. In summary, register shifts often produce multiple model-validation metric outliers simultaneously, none of them conclusive. Therefore, correct identification of the source of the problem usually requires tedious, residue-by-residue analysis of a map and crystal structure model by an experienced crystallographer (Croll *et al.*, 2021 ▸). In cases where lower resolution maps provide little help in model validation, the recently developed *conkit-validate* may be an option as it is based on a purely geometrical comparison of model-derived and AI-predicted intramolecular contacts and distances (Sánchez Rodríguez *et al.*, 2022 ▸).

In a recent publication I presented *checkMySequence*, a tool for the automated detection of register-shift errors in cryo-EM models (Chojnowski, 2022 ▸). The method is based on *findMySequence*, a protein sequence identification tool for crystallography and cryo-EM (Chojnowski *et al.*, 2022 ▸). The *checkMySequence* algorithm detects regions in the input model where an alternative sequence assignment is more plausible, indicating potential sequence-assignment issues. Here, I show that the same approach can be applied to the analysis of MX models using refined coordinates and standard, model-bias-corrected combined crystallographic 2*mF*_o_ − *DF*_c_ maps. I describe in detail five crystal structure models deposited in the PDB with register-shift errors that can unambiguously be detected using *checkMySequence* but would be difficult to identify automatically using other available model-validation metrics.

## 2. Materials and methods

### 2.1. Crystal structure benchmark set

For benchmarks, I selected crystal structure models of proteins, with or without nucleic acid components, solved at a resolution between 2.0 and 3.0 Å from the PDB. I considered only models deposited with corresponding diffraction data. Out of 68 955 models fulfilling these criteria as of 14 June 2022, for computational efficiency I randomly selected 10 000 structures and downloaded the corresponding atomic coordinates and amino-acid sequences in mmCIF and FASTA formats, respectively, from PDBe (Armstrong *et al.*, 2020 ▸). Maximum-likelihood Fourier coefficients for combined (2*mF*_o_ − *DF*_c_) and difference (*mF*_o_ − *DF*_c_) maps calculated using *REFMAC*5 (Murshudov *et al.*, 2011 ▸) and *DCC* (Yang *et al.*, 2016 ▸) were downloaded in MTZ format from the RCSB (Burley *et al.*, 2019 ▸).

### 2.2. Selection of test fragments

The performance of the sequence-assignment procedure implemented in *findMySequence* was tested using a large set of continuous, protein-chain test fragments. Randomly selecting three continuous fragments of 10 residues and three of 20 residues from each of the 13 525 unique protein chains in the benchmark set resulted in two sets of 40 575 test fragments each. Fragments with unknown residues, marked ‘UNK’ in the model, were rejected. Fragments for which the residue count did not match the difference between flanking residue numbers (possibly noncontinuous) were also rejected. This resulted in small differences between the expected and observed number of test fragments, which are 39 774 and 38 718 for 10 and 20 residues, respectively.

### 2.3. Data analysis and processing software

Benchmark-set structures were analysed fully automatically using *checkMySequence* version 1.4.1 and *findMySequence* version 1.0.8. Structural models with plausible sequence-register errors described in this work were analysed and rebuilt interactively using *Coot* version 0.9.8.4 and *CCP*4 version 8.0.005 within *CCP*4 Cloud version 1.7.006 (Krissinel *et al.*, 2022 ▸). Unless otherwise stated, corrected models were refined automatically using *REFMAC*5 version 5.8.0267 and *PDB-REDO* version 7.38 (Joosten *et al.*, 2014 ▸). Figures were prepared using *PyMOL* (DeLano, 2002 ▸) and *matplotlib* (Hunter, 2007 ▸). Structural superposition was performed using *GESAMT* version 1.18 (Krissinel, 2012 ▸) and the corresponding root-mean-square deviation (r.m.s.d.) values were calculated using C^α^ atoms. Map–model correlation coefficients were calculated using *EDSTATS* version 1.0.9 (Tickle *et al.*, 1998 ▸) and model geometry was analysed using *MolProbity*.

## 3. Results and discussion

### 3.1. Sequence-assignment statistics

The *checkMySequence* program systematically aligns continuous fragments of an input protein model to the target sequence based on the corresponding map. The program internally uses an algorithm implemented in *findMySequence* that scores each sequence alignment with a *p*-value: the probability that the alignment is observed by chance. Cases where the *p*-value is smaller than a predefined threshold and the new sequence alignment is different from the input model may indicate a register shift. I have recently shown (Chojnowski, 2022 ▸) that this approach can reliably identify register-shift errors in cryo-EM models. Unlike cryo-EM, however, MX electron-density maps are calculated using phase information derived from atomic models. This inevitably results in model bias and the presence of electron-density map features derived from the model and not from the experimental data, which may obscure errors. Although the model-bias issue is addressed with the maximum-likelihood maps commonly used for MX model building and interpretation, it was not clear whether and to what extent it would affect the performance of the AI-based classifier implemented in *findMySequence*. In particular, it was necessary to verify the choice of the *p*-value threshold previously defined for analysis of cryo-EM models in the context of MX models.

In the first step I analysed the distribution of *p*-values for test fragments randomly selected from benchmark structures as described in Section 2. The number of test fragments for which the reassigned and model sequences differed was relatively small: 389 out of 39 774 and 197 out of 38 718 test fragments of 10 and 20 residues, respectively. The number of test fragments with misassigned sequences is also significantly fewer than observed previously for EM structures (Chojnowski, 2022 ▸). This agrees with the estimated accuracy of residue-type classifiers used in *findMySequence*, which is noticeably higher for MX than for cryo-EM (Chojnowski *et al.*, 2022 ▸).

**Figure 1:**
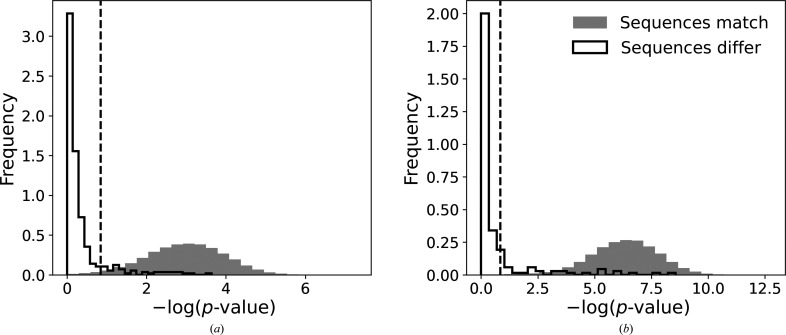
Statistics of sequence reassignment of randomly selected continuous protein chain fragments from benchmark-set MX structures. Grey and contoured histograms represent cases where newly assigned sequences match or differ, respectively, from the reference model for test fragments of (*a*) 10 and (*b*) 20 amino acids. The vertical dashed line depicts a standard threshold used by *checkMySequence* for outlier identification in cryo-EM models. The ordinate axes of the plots show −log(*p*-value); higher values correspond to lower *p*-values and more reliable sequence assignments. Frequency histograms are shown for clarity, but the sets presented in each panel are strongly unbalanced. The number of test fragments with reassigned sequences that do not match the reference model is 1% of the overall number of test fragments in the benchmark set.

Test fragments with correctly and incorrectly assigned sequences are clearly separated by the *p*-value, which is an indicator of the strong predictive power of the classifier (Fig. 1 ▸). For the sake of simplicity, the threshold defined previously for cryo-EM structures (a *p*-value of 0.14) was also used for the MX structures. In the current benchmark set of MX structures, this threshold corresponds to a 98.0% and 99.7% one-sided confidence interval for correct sequence assignment for fragments of 10 and 20 residues, respectively (Fig. 1 ▸). Moreover, fewer than 0.1% of test fragments were assigned an incorrect sequence with a *p*-value below this threshold (regardless of fragment length). Even though it is not known at this stage how many of these originate from structures with sequence-register issues, they correspond to model fragments that are very ‘unusual’ in statistical terms and thus deserve closer attention.

### 3.2. Benchmark-set analysis with *checkMySequence*

The *checkMySequence* program was used to systematically scan all of the crystal structures in the benchmark set, with the parameters derived in the previous section, deposited models and corresponding maximum-likelihood 2*mF*_o_ − *DF*_c_ maps. Analysis of the input structures took 18 s on average and less than 105 s for 99% of the tasks. Overall, the program identified sequence-assignment issues in 264 out of 10 000 structures from the benchmark set. They include 26 structures with residue-indexing issues, such as an unmodelled loop that was ignored in the residue numbering (no gap) and 86 structures with sequence mismatches or unidentified residues in a model. In 89 structures *checkMySequence* failed to assign to a reference sequence at least one protein chain consisting of ten or more amino acids. Given the high sensitivity of *findMy­Sequence*, which was used here to identify reference sequences, this may indicate chains that are very poorly resolved in the electron density (Chojnowski *et al.*, 2022 ▸). Finally, *checkMySequence* identified plausible register shifts in 70 structures. From these I selected five models, in which I corrected register-shift errors using interactive modelling software. They are presented in detail below.

### 3.3. Case study 1: WD40-repeat domain from *Thermomonospora curvata*

The WD40 repeats are a large family of proteins with a variable-size β-propeller fold. The WD40-repeat protein from *T. curvata* contains seven blades. The deposited crystal structure model (PDB entry 5yzv; Shen *et al.*, 2018 ▸), with five molecules in the asymmetric unit, was solved by molecular replacement (MR) with *Phaser* (McCoy *et al.*, 2007 ▸) and was refined to 2.5 Å resolution with *R*_work_ and *R*_free_ values of 0.225 and 0.259, respectively, and a clearly elevated clashscore of 27. Automated processing with *PDB-REDO* did not improve the validation scores (clashscore of 36 and *R*_work_ and *R*_free_ values of 0.199 and 0.259, respectively), indicating that the refinement strategy was not an issue here. The different molecules in the asymmetric unit have also a relatively large structural variability, reaching an r.m.s.d. of 1.2 Å, which is unexpected given the compact fold of the crystallized protein.

**Figure 2:**
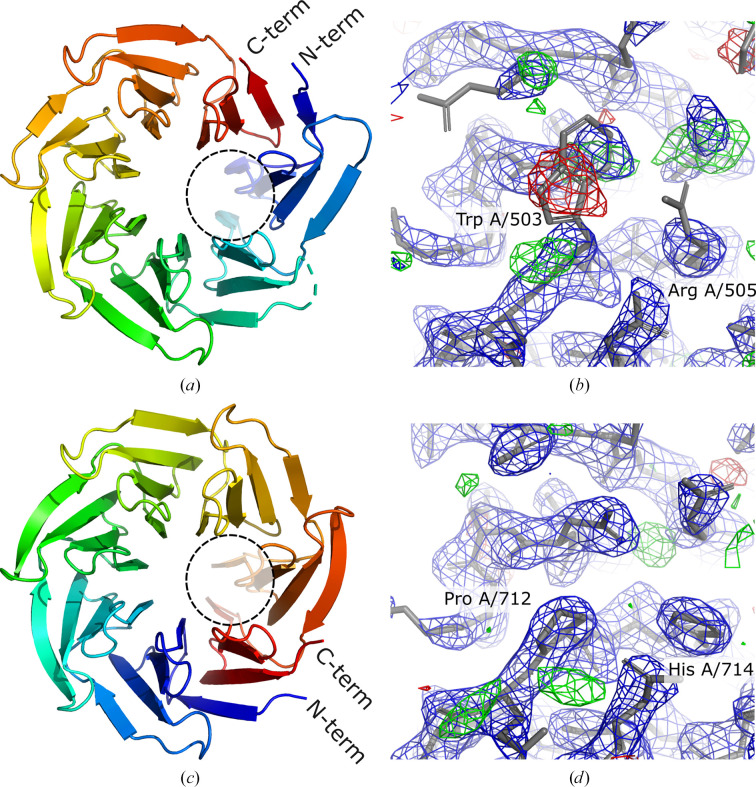
Comparison of deposited (*a*, *b*) and corrected (*c*, *d*) models of the WD40-repeat domain from *T. curvata*. Three molecules in the asymmetric unit of the deposited crystal structure were rotated about a sevenfold pseudo-symmetry axis of the structure (*a*, *c*), resulting in a number of clear density outliers in the model, for example Trp A/503 and Arg A/505 as labelled in (*b*). Correcting the molecule rotation results in a much better fit to the data (*d*). The model fragments shown in (*b*) and (*d*) are indicated by dashed circles in (*a*) and (*c*), respectively. The combined 2*mF*_o_ − *DF*_c_ (blue) and difference *mF*_o_ − *DF*_c_ (red/green) maximum-likelihood maps calculated using *REFMAC*5 are shown at 1.5σ and 3σ levels, respectively.

A *checkMySequence* analysis of the deposited coordinates revealed multiple register shifts in three of the five molecules in the asymmetric unit. The alternative sequences were assigned with a *p*-value below 0.01 and are therefore of high confidence (Fig. 1 ▸). However, the suggested register shifts were unusually large (up to 200 residues) and inconsistent between neighbouring chain fragments, even though no clear tracing issues were visible in the model. A more detailed inspection of the model and map revealed a number of side chains with strong difference-density peaks (for example Trp A/503 in Fig. 2 ▸ *b*), confirming that the structure may indeed suffer from an unusual modelling issue.

The source of the problem turned out to be a rotation about a sevenfold pseudo-symmetry axis of the protein, resulting in an inconsistent register of β-propeller blades in the model (Figs. 2 ▸ *a* and 2 ▸ *c*). The sequence differences between WD40-repeat blades were presumably obscured by the presence of an approximate sevenfold symmetry of the backbone during the MR search. This resulted in a partially incorrect sequence register of three molecules in the initial MR solution that was overlooked during the subsequent refinement steps. To confirm this, I corrected the model following suggestions from the *checkMySequence* analysis. As the refinement of register-shifted chains resulted in their deformation (chains *A*, *C* and *D* have an r.m.s.d. of over 1.0 Å when compared with chains with a correct register), I replaced them with a very reliable prediction from the AlphaFold Protein Structure Database (release v3 for UniProt entry P49695 with pLDDT > 90; Varadi *et al.*, 2022 ▸). To enforce a correct sequence register, I superposed the prediction onto the chains after reassigning the deposited model chains to a target sequence using *findMySequence*. Refinement of the corrected model using *REFMAC*5 with jelly-body restraints required 150 cycles to converge but resulted in notably better quality scores compared with the deposited coordinates; the *R*_work_ and *R*_free_ values were reduced to 0.170 and 0.204, respectively (from 0.225 and 0.259, respectively) and the clashscore was reduced to 4 (from 27). The other model-quality metrics also improved; for example, for chain *A* shown in Fig. 2 ▸ the map–model correlation coefficient increased from 0.89 from 0.96, the fraction of nonrotameric side chains decreased from 9.6 to 4.9% and the fraction of Ramachandran plot outliers decreased from 1.4% to zero. The refinement resulted in relatively small changes in the chains replaced with the initial *AlphaFold*2 model prediction (r.m.s.d. of 0.4 Å compared with the initial model). The overall quality of the map–model fit, however, improved noticeably (Fig. 2 ▸ *d*). In contrast to the deposited coordinates, the different WD40-repeat molecules in the final crystal structure model are also virtually identical, with r.m.s.d.s not exceeding 0.45 Å, which shows that the initially observed structural diversity was indeed a consequence of sequence misassignment.

Refinement of the WD40-repeat protein model with an incorrect sequence register resulted in multiple issues, including missing loops and backbone and side-chain geometry outliers. In principle, these could have been corrected using interactive software, but the predicted model turned out to be good enough to simply replace the existing model. It must be stressed, however, that this strategy may not be applicable to all cases as AI-based predictions often require substantial rebuilding to match experimental data (Terwilliger *et al.*, 2023 ▸).

### 3.4. Case study 2: *Helicobacter pylori* helicase with degraded helices

The structure of DnaB helicase from *H. pylori* (*Hp*DnaB) consists of two globular domains separated by a linker forming helices 7, 8 and 9. The crystallized *Hp*DnaB variant consists of a globular N-terminal domain (NTD) and helix 7. The deposited crystal structure model (PDB entry 3gxv; Kashav *et al.*, 2009 ▸) with two molecules in the asymmetric unit was solved by MR using *Phaser* with the N-terminal domain of a related helicase from *Mycobacterium tuberculosis* as a search model (PDB entry 2r5u; 25% sequence identify). The final model was refined at 2.5 Å resolution to reported *R*_work_ and *R*_free_ values of 0.249 and 0.278, respectively, with a clashscore of 32. Optimization of the refinement strategy with *PDB-REDO* reduced the clashscore to 4.57 at the expense of slightly worse *R*_work_ and *R*_free_ factors of 0.262 and 0.291, respectively.

The NTD and helix 7 dimer in the asymmetric unit is stabilized by two short, helical peptides, which the authors of the structure identified as helix 7 degraded from a complete construct. This was further confirmed by crystal electrophoresis and mass-spectrometry experiments. It is worth noting that alternatively to the interpretation of the authors the crystal content may be inhomogeneous, with a mixture of degraded helices 7 and additional complete *Hp*DnaB molecules with disordered NTDs in the asymmetric unit. This seems plausible given the crystal packing, the very high solvent content of the deposited crystal structure (72.4%) and the results of Matthews coefficient analysis, which suggests four *Hp*DnaB molecules (NTD and helix 7) in the asymmetric unit. This would explain the relatively high *R*_work_ and *R*_free_ factors of the deposited model.

**Figure 3:**
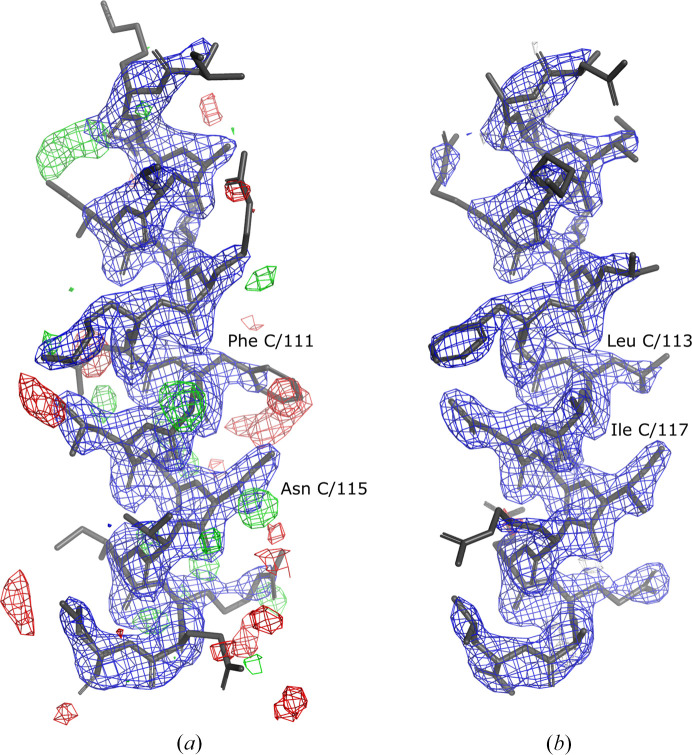
Crystal structure of an isolated helix 7 from the N-terminal domain (NTD) of *H. pylori* DnaB helicase (*Hp*DnaB). Comparison of deposited (*a*) and corrected (*b*) models. The peaks in the difference-density *mF*_o_ − *DF*_c_ map can be eliminated by shifting the model sequence register by two residues. For example, the clearly too large Phe C/111 resulting in a prominent negative (red) difference-density peak is replaced with a smaller Leu C/113 in the model with the corrected sequence register. Similarly, a clear positive peak (green) near Asn C/115 is interpreted as an Ile C/117 side chain in the corrected model. The combined 2*mF*_o_ − *DF*_c_ (blue) and difference *mF*_o_ − *DF*_c_ (red/green) maximum-likelihood maps calculated using *REFMAC*5 and *PDB_REDO* are shown at 2σ and 3σ levels, respectively. At this threshold no difference-density map features are visible in the presented asymmetric unit fragment of the corrected model.

One of the two isolated helices 7 in the crystal structure (chain *C*) shows a very prominent register shift in the *checkMySequence* analysis, with the alternative sequence-assignment *p*-value below 0.001 (Fig. 1 ▸). This is confirmed by the presence of strong difference-density peaks suggesting that several side chains are misaligned in the model, for example Phe C/111 and Asn C/115 (Fig. 3 ▸ *a*).

Automated refinement with *PDB-REDO* of the model with helix 7 (chain *C*) reassigned to the target sequence with *findMySequence* resulted in a much better fit of the coordinates to the corresponding 2*mF*_o_ − *DF*_c_ map and in a reduction of strong difference-density peaks (Fig. 3 ▸ *b*). This suggests that the new sequence register indeed fits the data better. Moreover, local quality scores for the model of helix 7 clearly improved. The map–model correlation coefficient increased from 0.81 to 0.91 and the fraction of nonrotameric side chains decreased from 16.7% to 0. Both the deposited and the corrected model have a single Ramachandran plot outlier: a poorly resolved residue at the N-terminus of helix 7. Correction of the model sequence resulted in a negligible reduction in the validation scores (*R*_work_ and *R*_free_ factors of 0.257 and 0.289, respectively, and a clashscore of 7.55). This, however, can be attributed to a relatively small (albeit important for model interpretation) modification of the coordinates (a difference in 18 out of over 2000 non-H atoms) and relatively high overall *R* values, as discussed above.

### 3.5. Case study 3: a hydrogenase from *Thermosipho melanesiensis*

**Figure 4:**
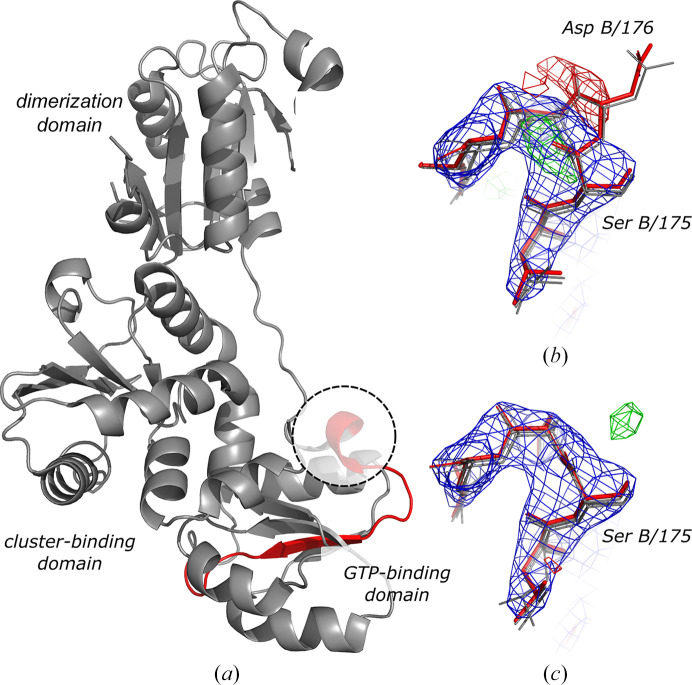
Crystal structure of HydF maturase with a register-shifted fragment of a GTP-binding domain shown in red (*a*). The dashed circle indicates the region of the deposited model shown in (*b*) with the corresponding electron-density maps. Asp B/176 is a clear map–model fit outlier that results in a strong negative peak in the difference-density map and a register shift in the fragment shown in red in (*a*). The remaining three HydF chains in the asymmetric unit superimposed onto chain *B* are shown in grey. After correcting the model and subsequent restrained refinement the map–model agreement clearly improves (*c*). The combined 2*mF*_o_ − *DF*_c_ (blue) and difference *mF*_o_ − *DF*_c_ (red/green) maximum-likelihood maps calculated using *REFMAC*5 and *PDB-REDO* are shown at 1.5σ and 3σ levels, respectively.

HydF is one of the maturation proteins that are required to activate an [FeFe] hydrogenase (HydA). The structure of *T. melanesiensis* HydF (*Tme*HydF) is composed of three domains (Fig. 4 ▸ *a*): dimerization (residues 7–166), GTP-binding (residues 172–262) and cluster-binding (residues 263–395) domains. The structure of the protein in complex with an Fe–S cluster (PDB entry 5kh0; Caserta *et al.*, 2017 ▸) was solved by MR using a closely related homologue, an apo HydF structure from *T. neapolitana*, as a search model (PDB entry 3qq5; 97% sequence identity). The deposited model with four molecules in the asymmetric unit was refined at 2.8 Å resolution to *R*_work_ and *R*_free_ values of 0.233 and 0.262, respectively, and a clashscore of 5.55. Automated refinement of the deposited model with *PDB-REDO* resulted in *R*_work_ and *R*_free_ values of 0.225 and 0.261, respectively, and a clashcore of 17.53.

An analysis with *checkMySequence* identified plausible register shifts with a *p*-value below 0.001 in a β-strand between residues 178 and 194 at the N-terminus of the GTP-binding domain in all four *Tme*HydF chains in the asymmetric unit (Fig. 4 ▸ *a*). Closer inspection revealed that the shift is caused by a clear insertion at residue 176 in chains *B* and *D* (Fig. 4 ▸ *b*) or a mistraced region between residues 180 and 182 in chains *A* and *C* (not shown). The insertions are further compensated by deletions at the same position in all four chains (residue 192). The small difference between the actual and reported residue span of the register-shifted region reflects the inherent accuracy of the algorithm implemented in *checkMySequence*, which is within five residues (Chojnowski, 2022 ▸). The register-shifted region does not affect the conformation of the active site of the protein in the cluster-binding domain, which was analysed in more detail by the authors of the structure. After correcting the register-shifted fragments with *findMySequence* and *Coot*, and subsequent restrained refinement in *PDB-REDO* with* REFMAC*5, the *R*_work_ and *R*_free_ improved slightly to 0.221 and 0.252, respectively (from 0.225 and 0.261) and the clashscore decreased to 11 from 17. Moreover, the clearly visible, prominent difference-density peaks in regions corresponding to incorrectly assigned side chains disappeared (Fig. 4 ▸ *c*). The local map–model correlation coefficient for the register-shifted fragment increased from 0.91 to 0.96 and the fraction of nonrotameric side chains decreased from 23.1 to 7.7%. An unusually high number of Ramachandran plot outliers (13.3%) in the register-shifted region decreased to zero in the corrected model.

The *Tme*HydF structure prediction downloaded from the AlphaFold Protein Structure Database (release v3 for UniProt entry A6LMQ7) has a different orientation of the dimerization domain relative to the remaining two domains. As the uncertainty of the relative domain orientation is not reflected in the Predicted Alignment Error (PAE) plot, the use of the *AlphaFold*2 prediction for model building or as an MR search model would be not straightforward. The predicted and corrected crystal structure models, however, agree very well locally. For example, the GTP-binding domains superpose with an r.m.s.d. of 0.78 Å, with the only significant differences in a loop following the register-shifted region (predicted with low accuracy; pLDDT < 50). Thus, the prediction could in principle be used to identify and correct the register-shift error in the deposited model.

### 3.6. Case study 4: protein L31e from the large ribosomal subunit of *Haloarcula marismortui*

**Figure 5:**
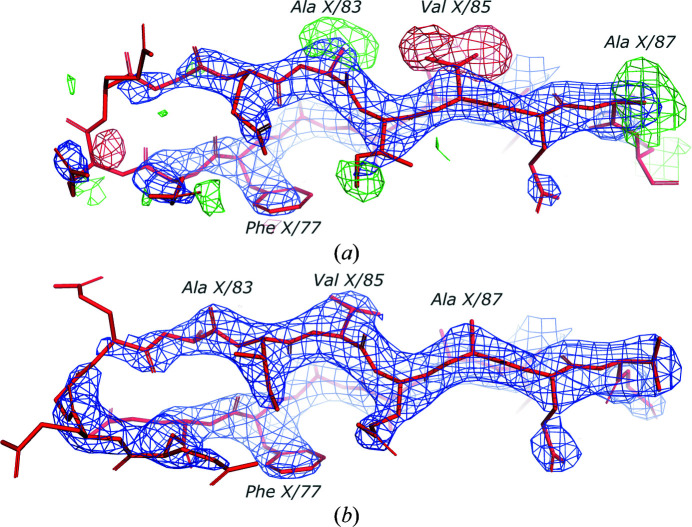
Ribosomal protein L31e from the crystal structure of a large ribosomal subunit from *H. marismortui*. A solvent-exposed, poorly resolved loop following Phe X/77 was traced too short in the original model and resulted in a two-residue sequence-register shift in a C-terminal part of the chain. Strong difference-density peaks showing a few excess and missing atoms in the deposited structures for Ala X/83, Val X/85 and Ala X/87 (*a*) disappear after refining the model with the corrected sequence register (*b*). The combined 2*mF*_o_ − *DF*_c_ (blue) and difference *mF*_o_ − *DF*_c_ (red/green) maximum-likelihood maps calculated using *REFMAC*5 and *PDB-REDO* are shown at 1.5σ and 3σ levels, respectively.

The crystal structure of the 50S large ribosomal subunit of *H. marismortui* has been determined at 2.65 Å resolution and refined to *R*_work_ and *R*_free_ values of 0.176 and 0.214, respectively, and a clashscore of 16 (PDB entry 1yi2; Tu *et al.*, 2005 ▸). An analysis with *checkMySequence* revealed that a C-terminal fragment of a peripheral ribosomal protein L31e may be shifted by two residues (*p*-value 0.1) between residues X/77 and X/88 (the last modelled residue in the chain). A closer inspection of the deposited model and maps revealed several difference-density peaks in the C-terminal fragment of the protein (Fig. 5 ▸ *a*). After reassigning the fragment to the target sequence with *findMySequence*, rebuilding a short loop preceding it in *Coot* and subsequent refinement with *PDB-REDO*, the overall map–model agreement of the chain clearly improved (Fig. 5 ▸ *b*). The final model *R*_work_ and *R*_free_ did not change compared with the values obtained using *PDB-REDO* for the deposited coordinates (0.166 and 0.206, respectively, versus 0.167 and 0.206) and the clashscore decreased slightly from 4.33 to 3.29. The local map–model correlation coefficient for the register-shifted fragment starting at Phe 77/X increased to 0.92 from 0.89 and the fraction of nonrotameric side chains decreased from 22.2% to zero. Interestingly, the register-shifted region also had an unusually high number of Ramachandran plot outliers of 20%, which decreased to zero in the corrected model.

A structure prediction from the AlphaFold2 database (release v3 for UniProt entry P18138) has overall high confidence and agrees very well with the corrected crystal structure model (r.m.s.d. of 0.47 Å including the poorly resolved loop that had to be rebuilt). Although the loop was scored slightly lower than the remaining structure (pLDDT of between 80 and 85), it could be directly used for interpretation of the poorly resolved map region and to avoid the register shift in the model.

### 3.7. Case study 5: a glutaminase from *Geobacillus kaustophilus*

**Figure 6:**
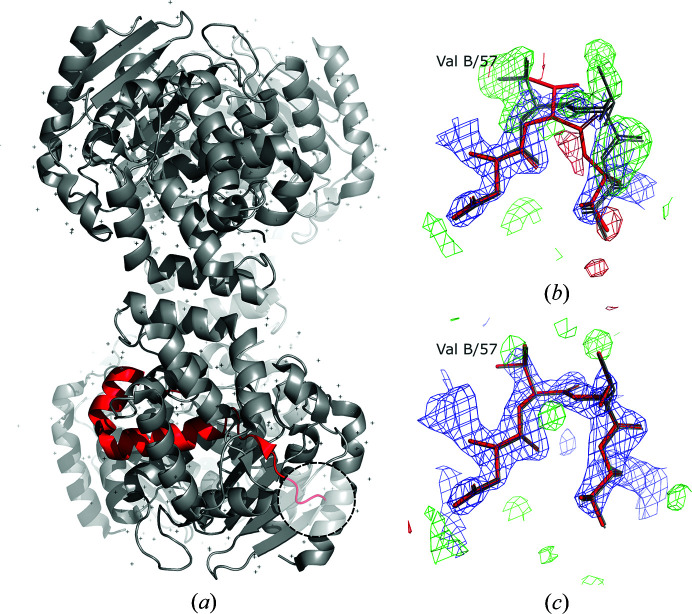
Crystal structure model of a putative glutaminase from *G. kaustophilus* (*a*). The dashed circle indicates the region of the deposited model shown in (*b*) with the corresponding maps. A deletion near Val B/57 results in multiple strong positive difference-density map peaks and a register shift in the fragment shown in red in (*a*). The panel depicts residues 56–59 from chain *B* (red) and superposed models of the remaining chains *A*, *C* and *D* (grey). After correcting the model and subsequent restrained refinement the map–model agreement clearly improves (*c*). The combined 2*mF*_o_ − *DF*_c_ (blue) and difference *mF*_o_ − *DF*_c_ (red/green) maximum-likelihood maps calculated using *REFMAC*5 and *PDB-REDO* are shown at 2σ and 3σ levels, respectively.

The structure of a glutaminase from *G. kaustophilus* was refined at 2.1 Å resolution with four molecules in the asymmetric unit (PDB entry 2pby) to *R*_work_ and *R*_free_ values of 0.195 and 0.249, respectively, and a clashscore of 7.62, which decreased to 0.174 and 0.207, respectively, and a clashscore of 6.44 after automated refinement-strategy optimization with *PDB-REDO*. The *checkMySequence* analysis revealed an unambiguous shift of sequence register in chain *B* between residues B/54 and B/92 (*p*-value of less than 0.0001). Model inspection revealed an insertion at residue B/57 starting a register shift that continued until a chain break at residue B/92 (Figs. 6 ▸ *a* and 6 ▸ *b*). As the structure remains unpublished, little is known about the structure-determination details. According to the PDB file header it was solved by MR using *EPMR* (Kissinger *et al.*, 2001 ▸) with the structure of a related glutaminase from *Bacillus subtilis* (PDB entry 1mki) as the search model. The two structure models are very similar, and 275 out of the 291 and 321 residues in the target and search models, respectively, align with an r.m.s.d. of 1.1 Å. Nevertheless, they share only 46% sequence identity and have multiple loops of different lengths and/or conformations, which suggests that the deposited model underwent an extensive (possibly automated) rebuilding that may have resulted in the register shift. All four chains in the model are virtually identical (r.m.s.d. of 0.23 Å), with the only visible difference being in the conformation of residues flanking a solvent-exposed, disordered loop between residues 92 and 109, and the clearly visible deletion in chain *B* that resulted in a register shift (Fig. 6 ▸ *a*).

After correcting the main-chain tracing issue in chain *B*, reassigning the register-shifted model fragment to the sequence using *findMySequence* and subsequent automated refinement using *REFMAC*5 and *PDB-REDO*, the *R*_work_ and *R*_free_ values decreased to 0.168 and 0.197, respectively, and the clashscore decreased to 1.90 (from values of 0.174, 0.207 and 6.44, respectively, after initial *PDB-REDO* optimization). In addition, a better map–model fit was obtained for the affected residue range B/54–92 (map–model correlation coefficient of 0.96 versus 0.89), as well as a better agreement between all four molecules in the asymmetric unit (Fig. 6 ▸ *c*). Within the corrected residue range B/54–92 the fraction of nonrotameric side chains and Ramachandran plot outliers (24.2% and 5.4%, respectively) both decreased to zero in the corrected model.

## 4. Conclusions

The purpose of building scientific models is to enable the interpretation of complex experimental data in the light of the available theoretical knowledge. Consequently, models are always provisional and can be updated if new evidence becomes available. This applies to the structural models of macromolecules; they are tentative and can be always improved, with better data, by a laborious iterative refinement or with new, more robust data-analysis software.

Here, I have presented *checkMySequence*, a fast and fully automated method for the identification of register shifts in crystal structure models of proteins. I showed that it can identify errors in structural models that were already considered to be ‘good enough’ and deposited in the PDB. The sequence-assignment issues that I have selected for detailed description do not affect the conclusions derived from the corresponding models by their authors as they were found in peripheral regions (ribosomal protein L31e), affect the overall model quality (WD40-repeat) or affect only one of multiple protein copies in the asymmetric unit (glutaminase). It is probably for this reason that they went unnoticed in the first place. It is not clear, however, how these errors affected or will affect subsequent studies. It also remains to be seen how many models deposited in the PDB have register errors that affected their functional analysis. This cannot be studied *en masse* as it requires an individual approach by specialists, either revisiting their own models or aggregating available structural data. For others a simple warning about a potential sequence-assignment issue in a PDB-deposited structure can help to avoid problems. I believe that *checkMySequence* will prove helpful with all of them.

Although all of the described issues could have been deduced from the presence of prominent difference-density peaks, unusual backbone geometry, a reduced map–model correlation coefficient, local differences between different copies of the same molecule in the asymmetric unit, high clashscore or elevated *R* factors, only *checkMySequence* clearly annotated the errors. This should make *checkMy­Sequence* particularly useful for inexperienced users or when validated models are very large, such as the 50S ribosomal subunit presented above, where a detailed residue-by-residue analysis of the map–model fit and model geometry is not feasible.

I have also shown that a few of the presented errors can be corrected (and possibly could have been avoided) using AI-based predictions for structure determination, as has already become a standard. In some cases, however, this would not be enough to avoid an error; for example, when an isolated fragment needs to be assigned to a target sequence (as in the case of *Hp*DnaB) or when an error is obscured by an unusual fold of a protein (WD40-repeat). In such cases *checkMy­Sequence* will prove to be especially useful.

The presented results were restricted to crystal structures determined between 2.0 and 3.0 Å resolution, which dominate the MX structures deposited in the PDB. In this resolution range all map–model fit problems are usually clearly visible in a map if properly presented, but are difficult to detect using an automatic algorithm. It is also relatively easy to present visual evidence that the new model does indeed better explain the experimental data. At lower resolutions this becomes increasingly difficult. Therefore, a more challenging analysis of register errors in low-resolution crystal structures, which are probably far more frequent, I leave for future collaborative work and a more robust methodology involving an approach restricted to model-geometry analysis that has been shown to complement *checkMy­Sequence* in poorly resolved cryo-EM map regions (Sánchez Rodríguez *et al.*, 2022 ▸).

## 5. Data and code availability

The latest version of the *checkMySequence* source code and the installation instructions are available at https://gitlab.com/gchojnowski/checkmysequence. The corrected models that are described here are available at https://doi.org/10.5281/zenodo.7650180.

## References

[bb1] Armstrong, D. R., Berrisford, J. M., Conroy, M. J., Gutmanas, A., Anyango, S., Choudhary, P., Clark, A. R., Dana, J. M., Deshpande, M., Dunlop, R., Gane, P., Gáborová, R., Gupta, D., Haslam, P., Koča, J., Mak, L., Mir, S., Mukhopadhyay, A., Nadzirin, N., Nair, S., Paysan-Lafosse, T., Pravda, L., Sehnal, D., Salih, O., Smart, O., Tolchard, J., Varadi, M., Svobodova-Vařeková, R., Zaki, H., Kleywegt, G. J. & Velankar, S. (2020). *Nucleic Acids Res.* **48**, D335–D343. 10.1093/nar/gkz990 PMC7145656 31691821

[bb2] Baek, M., DiMaio, F., Anishchenko, I., Dauparas, J., Ovchinnikov, S., Lee, G. R., Wang, J., Cong, Q., Kinch, L. N., Schaeffer, R. D., Millán, C., Park, H., Adams, C., Glassman, C. R., DeGiovanni, A., Pereira, J. H., Rodrigues, A. V., van Dijk, A. A., Ebrecht, A. C., Opperman, D. J., Sagmeister, T., Buhlheller, C., Pavkov-Keller, T., Rathina­swamy, M. K., Dalwadi, U., Yip, C. K., Burke, J. E., Garcia, K. C., Grishin, N. V., Adams, P. D., Read, R. J. & Baker, D. (2021). *Science*, **373**, 871–876.

[bb3] Brünger, A. T. (1992). *Nature*, **355**, 472–475. 10.1038/355472a0 18481394

[bb4] Brzezinski, D., Dauter, Z., Minor, W. & Jaskolski, M. (2020). *FEBS J.* **287**, 2685–2698. 10.1111/febs.15314 PMC7340579 32311227

[bb5] Burley, S. K., Berman, H. M., Bhikadiya, C., Bi, C., Chen, L., Di Costanzo, L., Christie, C., Dalenberg, K., Duarte, J. M., Dutta, S., Feng, Z., Ghosh, S., Goodsell, D. S., Green, R. K., Guranović, V., Guzenko, D., Hudson, B. P., Kalro, T., Liang, Y., Lowe, R., Namkoong, H., Peisach, E., Periskova, I., Prlić, A., Randle, C., Rose, A., Rose, P., Sala, R., Sekharan, M., Shao, C., Tan, L., Tao, Y., Valasatava, Y., Voigt, M., Westbrook, J., Woo, J., Yang, H., Young, J., Zhuravleva, M. & Zardecki, C. (2019). *Nucleic Acids Res.* **47**, D464–D474. 10.1093/nar/gky1004 PMC6324064 30357411

[bb6] Casañal, A., Lohkamp, B. & Emsley, P. (2020). *Protein Sci.* **29**, 1055–1064. 10.1002/pro.3791 PMC7096722 31730249

[bb7] Caserta, G., Pecqueur, L., Adamska-Venkatesh, A., Papini, C., Roy, S., Artero, V., Atta, M., Reijerse, E., Lubitz, W. & Fontecave, M. (2017). *Nat. Chem. Biol.* **13**, 779–784. 10.1038/nchembio.2385 28553946

[bb8] Chojnowski, G. (2022). *Acta Cryst.* D**78**, 806–816. 10.1107/S2059798322005009 PMC9248842 35775980

[bb9] Chojnowski, G., Simpkin, A. J., Leonardo, D. A., Seifert-Davila, W., Vivas-Ruiz, D. E., Keegan, R. M. & Rigden, D. J. (2022). *IUCrJ*, **9**, 86–97. 10.1107/S2052252521011088 PMC8733886 35059213

[bb10] Croll, T. I., Williams, C. J., Chen, V. B., Richardson, D. C. & Richardson, J. S. (2021). *Biophys. J.* **120**, 1085–1096. 10.1016/j.bpj.2020.12.029 PMC7834719 33460600

[bb11] DeLano, W. L. (2002). *CCP4 Newsl. Protein Crystallogr.* **40**, 82–92.

[bb12] Gore, S., Sanz García, E., Hendrickx, P. M. S., Gutmanas, A., Westbrook, J. D., Yang, H., Feng, Z., Baskaran, K., Berrisford, J. M., Hudson, B. P., Ikegawa, Y., Kobayashi, N., Lawson, C. L., Mading, S., Mak, L., Mukhopadhyay, A., Oldfield, T. J., Patwardhan, A., Peisach, E., Sahni, G., Sekharan, M. R., Sen, S., Shao, C., Smart, O. S., Ulrich, E. L., Yamashita, R., Quesada, M., Young, J. Y., Nakamura, H., Markley, J. L., Berman, H. M., Burley, S. K., Velankar, S. & Kleywegt, G. J. (2017). *Structure*, **25**, 1916–1927.

[bb13] Hooft, R. W. W., Vriend, G., Sander, C. & Abola, E. E. (1996). *Nature*, **381**, 272. 10.1038/381272a0 8692262

[bb14] Hunter, J. D. (2007). *Comput. Sci. Eng.* **9**, 90–95.

[bb15] Jones, D. T. & Thornton, J. M. (2022). *Nat. Methods*, **19**, 15–20. 10.1038/s41592-021-01365-3 35017725

[bb16] Joosten, R. P., Long, F., Murshudov, G. N. & Perrakis, A. (2014). *IUCrJ*, **1**, 213–220. 10.1107/S2052252514009324 PMC4107921 25075342

[bb17] Jumper, J., Evans, R., Pritzel, A., Green, T., Figurnov, M., Ronneberger, O., Tunyasuvunakool, K., Bates, R., Žídek, A., Potapenko, A., Bridgland, A., Meyer, C., Kohl, S. A. A., Ballard, A. J., Cowie, A., Romera-Paredes, B., Nikolov, S., Jain, R., Adler, J., Back, T., Petersen, S., Reiman, D., Clancy, E., Zielinski, M., Steinegger, M., Pacholska, M., Berghammer, T., Bodenstein, S., Silver, D., Vinyals, O., Senior, A. W., Kavukcuoglu, K., Kohli, P. & Hassabis, D. (2021). *Nature*, **596**, 583–589. 10.1038/s41586-021-03819-2 PMC8371605 34265844

[bb18] Kashav, T., Nitharwal, R., Abdulrehman, S. A., Gabdoulkhakov, A., Saenger, W., Dhar, S. K. & Gourinath, S. (2009). *PLoS One*, **4**, e7515. 10.1371/journal.pone.0007515 PMC2761005 19841750

[bb19] Kissinger, C. R., Gehlhaar, D. K., Smith, B. A. & Bouzida, D. (2001). *Acta Cryst.* D**57**, 1474–1479. 10.1107/s0907444901012458 11567162

[bb20] Krissinel, E. (2012). *J. Mol. Biochem.* **1**, 76–85. PMC5117261 27882309

[bb21] Krissinel, E., Lebedev, A. A., Uski, V., Ballard, C. B., Keegan, R. M., Kovalevskiy, O., Nicholls, R. A., Pannu, N. S., Skubák, P., Berrisford, J., Fando, M., Lohkamp, B., Wojdyr, M., Simpkin, A. J., Thomas, J. M. H., Oliver, C., Vonrhein, C., Chojnowski, G., Basle, A., Purkiss, A., Isupov, M. N., McNicholas, S., Lowe, E., Triviño, J., Cowtan, K., Agirre, J., Rigden, D. J., Uson, I., Lamzin, V., Tews, I., Bricogne, G., Leslie, A. G. W. & Brown, D. G. (2022). *Acta Cryst.* D**78**, 1079–1089.

[bb22] Laskowski, R. A., MacArthur, M. W., Moss, D. S. & Thornton, J. M. (1993). *J. Appl. Cryst.* **26**, 283–291.

[bb23] McCoy, A. J., Grosse-Kunstleve, R. W., Adams, P. D., Winn, M. D., Storoni, L. C. & Read, R. J. (2007). *J. Appl. Cryst.* **40**, 658–674. 10.1107/S0021889807021206 PMC2483472 19461840

[bb24] Murshudov, G. N., Skubák, P., Lebedev, A. A., Pannu, N. S., Steiner, R. A., Nicholls, R. A., Winn, M. D., Long, F. & Vagin, A. A. (2011). *Acta Cryst.* D**67**, 355–367. 10.1107/S0907444911001314 PMC3069751 21460454

[bb25] Paysan-Lafosse, T., Blum, M., Chuguransky, S., Grego, T., Pinto, B. L., Salazar, G., Bileschi, M., Bork, P., Bridge, A., Colwell, L., Gough, J., Haft, D., Letunić, I., Marchler-Bauer, A., Mi, H., Natale, D., Orengo, C., Pandurangan, A., Rivoire, C., Sigrist, C. J. A., Sillitoe, I., Thanki, N., Thomas, P. D., Tosatto, S. C. E., Wu, C. & Bateman, A. (2022). *Nucleic Acids Res.* **51**, D418–D427. 10.1093/nar/gkac993 PMC9825450 36350672

[bb26] Prisant, M. G., Williams, C. J., Chen, V. B., Richardson, J. S. & Richardson, D. C. (2020). *Protein Sci.* **29**, 315–329. 10.1002/pro.3786 PMC6933861 31724275

[bb27] Read, R. J. (1986). *Acta Cryst.* A**42**, 140–149.

[bb28] Sánchez Rodríguez, F., Chojnowski, G., Keegan, R. M. & Rigden, D. J. (2022). *Acta Cryst.* D**78**, 1412–1427. 10.1107/S2059798322010415 PMC9716559 36458613

[bb29] Shen, C., Du, Y., Qiao, F., Kong, T., Yuan, L., Zhang, D., Wu, X., Li, D. & Wu, Y.-D. (2018). *Sci. Rep.* **8**, 12965. 10.1038/s41598-018-31140-y PMC6113231 30154510

[bb30] Sobolev, O. V., Afonine, P. V., Moriarty, N. W., Hekkelman, M. L., Joosten, R. P., Perrakis, A. & Adams, P. D. (2020). *Structure*, **28**, 1249–1258. 10.1016/j.str.2020.08.005 PMC7642142 32857966

[bb31] Terwilliger, T. C., Afonine, P. V., Liebschner, D., Croll, T. I., McCoy, A. J., Oeffner, R. D., Williams, C. J., Poon, B. K., Richardson, J. S., Read, R. J. & Adams, P. D. (2023). *Acta Cryst.* D**79**, 234–244. 10.1107/S205979832300102X PMC9986801 36876433

[bb32] Tickle, I. J., Laskowski, R. A. & Moss, D. S. (1998). *Acta Cryst.* D**54**, 243–252. 10.1107/s090744499701041x 9761889

[bb33] Tu, D., Blaha, G., Moore, P. B. & Steitz, T. A. (2005). *Cell*, **121**, 257–270. 10.1016/j.cell.2005.02.005 15851032

[bb34] Varadi, M., Anyango, S., Deshpande, M., Nair, S., Natassia, C., Yordanova, G., Yuan, D., Stroe, O., Wood, G., Laydon, A., Žídek, A., Green, T., Tunyasuvunakool, K., Petersen, S., Jumper, J., Clancy, E., Green, R., Vora, A., Lutfi, M., Figurnov, M., Cowie, A., Hobbs, N., Kohli, P., Kleywegt, G., Birney, E., Hassabis, D. & Velankar, S. (2022). *Nucleic Acids Res.* **50**, D439–D444. 10.1093/nar/gkab1061 PMC8728224 34791371

[bb35] Williams, C. J., Headd, J. J., Moriarty, N. W., Prisant, M. G., Videau, L. L., Deis, L. N., Verma, V., Keedy, D. A., Hintze, B. J., Chen, V. B., Jain, S., Lewis, S. M., Arendall, W. B., Snoeyink, J., Adams, P. D., Lovell, S. C., Richardson, J. S. & Richardson, J. S. (2018). *Protein Sci.* **27**, 293–315. 10.1002/pro.3330 PMC5734394 29067766

[bb36] Wlodawer, A., Dauter, Z., Porebski, P. J., Minor, W., Stanfield, R., Jaskolski, M., Pozharski, E., Weichenberger, C. X. & Rupp, B. (2018). *FEBS J.* **285**, 444–466. 10.1111/febs.14320 PMC5799025 29113027

[bb37] wwPDB Consortium (2018). *Nucleic Acids Res.* **47**, D520–D528. 10.1093/nar/gky949 PMC6324056 30357364

[bb38] Yang, H., Peisach, E., Westbrook, J. D., Young, J., Berman, H. M. & Burley, S. K. (2016). *J. Appl. Cryst.* **49**, 1081–1084. 10.1107/S1600576716004428 PMC4886994 27275151

